# Quantitative Proteomics and Lipidomics Analysis of Endoplasmic Reticulum of Macrophage Infected with *Mycobacterium tuberculosis*

**DOI:** 10.1155/2015/270438

**Published:** 2015-02-16

**Authors:** Najmuddin Mohd Saquib, Shilpa Jamwal, Mukul Kumar Midha, Hirdya Narain Verma, Venkatasamy Manivel

**Affiliations:** ^1^Immunology Group, International Centre for Genetic Engineering and Biotechnology, Aruna Asaf Ali Marg, New Delhi 110067, India; ^2^School of Life Sciences, Jaipur National University, Jaipur 302025, India; ^3^Drug Discovery Research Centre, Translational Health Science & Technology Institute, Gurgaon 122016, India

## Abstract

Even though endoplasmic reticulum (ER) stress associated with mycobacterial infection has been well studied, the molecular basis of ER as a crucial organelle to determine the fate of *Mtb* is yet to be established. Here, we have studied the ability of *Mtb* to manipulate the ultrastructural architecture of macrophage ER and found that the ER-phenotypes associated with virulent (H37Rv) and avirulent (H37Ra) strains were different: a rough ER (RER) with the former against a smooth ER (SER) with the later. Further, the functional attributes of these changes were probed by MS-based quantitative proteomics (133 ER proteins) and lipidomics (8 phospholipids). Our omics approaches not only revealed the host pathogen cross-talk but also emphasized how precisely *Mtb* uses proteins and lipids in combination to give rise to characteristic ER-phenotypes. H37Ra-infected macrophages increased the cytosolic Ca^2+^ levels by attenuating the ATP2A2 protein and simultaneous induction of PC/PE expression to facilitate apoptosis. However, H37Rv inhibited apoptosis and further controlled the expression of EST-1 and AMRP proteins to disturb cholesterol homeostasis resulting in sustained infection. This approach offers the potential to decipher the specific roles of ER in understanding the cell biology of mycobacterial infection with special reference to the impact of host response.

## 1. Introduction

Tuberculosis (TB) continues to haunt as a major health concern, and its spread has been aggravated by the onset of multidrug-resistant strains of* Mycobacterium tuberculosis* (*Mtb*). TB, one of the oldest reemerging diseases, remains to be eliminated. In 2012, TB worldwide accounted for 8.6 million cases and 1.3 million deaths from TB among HIV-negative people. Furthermore, an additional 0.32 million deaths were accounted for HIV-TB coinfection [1]. However, in spite of this endemic occurrence, the underlying mechanisms of pathogenesis of TB are not fully delineated. To deal with this issue, it is essential to identify newer ways to eliminate intracellular mycobacteria in multidrug-resistant TB infected individuals and to come up with highly potent therapeutic processes for treating such TB infected individuals.

Apoptosis is a cellular decision that involves the synthesis of a succession of cellular proteins to elicit an apoptotic signal. Although apoptosis in microbial infections leads to tissue damage, the initiation of apoptosis may be advantageous to the host, as it also ends up eliminating the microorganisms [2]. Macrophages play a critical role in the protection against mycobacterial infections [3].* Mtb* emerges as one of the highly successful human pathogens, due to its ability to survive by manipulating host cells by manipulation of multiple pathways [4–6]. Recent studies have revealed that macrophages employ apoptosis as an innate defense response against* Mtb* [6]. However, virulent* Mtb* does not elicit macrophage apoptosis and instead initiates necrosis at excessive intracellular bacterial cargoes [7]. Lim et al. have shown that the endoplasmic reticulum (ER) stress response may be linked to* Mtb*-induced apoptosis, and this process has an important role in controlling the survival of intracellular* Mtb* [4].

The ER has an essential role in folding secretory and cellular proteins during their transit, and ER chaperone proteins avert the toxic buildup of incorrectly folded secretory proteins. In addition to this critical role in protein folding, quality control, and targeting, the ER is also involved in the synthesis of a wide range of cellular lipids [8, 9] along with regulation of Ca^2+^ homeostasis [10]. Any malfunction in ER can lead to cell death by activating a series of ER chaperones that participate in the regulation of protein folding and the induction of cell death [11]. Even though apoptosis favoring the host protection is inferred and normally considered to be responsible for the modulation of intracellular* Mtb* survival, the biological significance of this event remains to be clarified.

Choi et al. showed ESAT-6 antigen stimulates ER stress-mediated apoptosis in* Mtb* infected macrophages that induce growth arrest by DNA damage-induced gene-153 (GADD153) production. Silencing of GADD153 increases intracellular bacillary loads [12]. Seimon et al. [13] using TB granuloma macrophages demonstrated that ER stress is induced in areas where apoptotic cells concentrate. Collectively these results imply that ER stress responses play important roles in TB pathogenesis.

Sohn et al. recently showed that Heparin-binding haemagglutinin antigen (HBHA) enters macrophages and stimulates apoptosis by effecting loss of mitochondrial transmembrane potential with concomitant reactive oxygen species (ROS) generation [14]. However, Choi et al. demonstrated that HBHA induces ROS production by the disruption of intracellular Ca^2+^ homeostasis. The ROS stimulate excess of proinflammatory cytokines thereby leading to ER stress-induced apoptosis [15].

More recently Jamwal et al. performed differential mitochondrial proteomics on human macrophages that were infected with either the avirulent* Mtb* strain H37Ra or its virulent counterpart H37Rv to identify several host proteins as virulence factors that induce either apoptosis in H37Ra infected macrophages or survival in H37Rv infected host cells. These proteins were capable of differentially influencing several biochemical pathways such as ATP production (ATP50 subunit), citric acid cycle (UQCRH and DLD) and associated electron transport chain, voltage-dependent anion channels (VDAC2), ROS (PRDX1), NO production (OAS2, SQRDL), phospholipid synthesis (ACSL1, ACSL4, and ACAT1), and fatty acid metabolism (HADHA), and lipid bodies (LBs) synthesis eventually leading to foamy macrophages formation in H37Rv infected macrophages [16].

The present study is designed to monitor the changes brought about by* Mtb* at the ER organelle level by observing the ultrastructural features of the host cell employing transmission electron microscopy to probe for alterations in morphology of the ER, as a function of the duration of infection. The basis behind this is that, by this comparison between the consequences of a virulent versus an avirulent infection, it should be possible to identify those functional changes that would contribute towards eventual survival and persistence of the intracellular pathogen.

*Mtb* productively infects macrophages by upsetting the maturation of its phagosome, generating an intracellular compartment with endosomal rather than lysosomal characteristics [17]. Ca^2+^ plays a significant role in different apoptotic pathways and is responsible for the phagosome-lysosome fusion [18]. ER is the primary site for maintaining cellular calcium homeostasis, decisions on cell survival versus death, and all processes involved in maintaining homeostasis of the various molecular constituents of the cell [19]. Therefore, ER can be one of the targets of mycobacterial manipulation in the macrophage. Accordingly, our analysis is directed at the perturbations induced in the ER proteome and lipidome of infected cells. Here again it was decided to compare between the effects of a virulent versus an avirulent infection so as to distinguish those effects that specifically relate to the virulence properties of the pathogen. For proteomic studies we employed the technique of stable isotope labeling of amino acids in cell culture (SILAC) wherein macrophages were first equilibrated with isotopically labeled lysine prior to infection [20]. This has enabled a quantitative comparison of the ER proteome by 2D-LC-MS/MS. For lipidomics study we employed shotgun lipidomics approach to quantitate the ER lipidome from the organic phase of the crude extract based on their distinct headgroups released upon MS/MS tandem mass spectrometry [21]. It is expected that such a comparison would provide mechanistic information on the nature of crosstalk between the host and the pathogen.

## 2. Materials and Methods

### 2.1. Cell Lines and Culture Conditions

THP-1 is a human monocyte leukemia cell line which was derived from a 1-year-old boy with acute monocytic leukemia [22]. THP-1 cells were cultured in RPMI 1640 supplemented with 10% FBS at a density of 2–10 × 10^5^ cells/mL at 37°C in a humidified, 5% CO_2_ atmosphere. Forty-eight hours before* Mtb* infection cells were seeded in a T-175 flask at 20–25 × 10^6^ cells per flask per 30 mL and differentiated using 30 ng of PMA washed with RPMI without serum and medium was replenished.

### 2.2. Infection of THP-1 Cell Line

Two strains of* Mtb*, namely, H37Ra and H37Rv, were used for the study. All experiments with live* Mtb* were performed in a Biosafety Level III containment lab. Tuberculosis Aerosol Challenge Facility (TACF) is equipped with state-of-the-art infrastructure for safe handling of pathogenic tubercle bacilli. THP-1 cells were infected with designated* Mtb* strains at the multiplicity of infection (MOI) of ~10. Experiments were set with THP-1 cells maintained till 4–8th passages. THP-1 cells were seeded at density of 25 × 10^6^/30 mL of T-175 flask or 10 × 10^6^/12 mL of 6-well plate.* Mtb* culture for infection purposes was prepared by spinning the bacterial culture at 3000 rpm/10 min, followed by resuspension of pellets in RPMI without FBS. Suspension was passed through series of needles, that is, 23 gauge, 26 gauge, and 30 gauge, in successive order to break the bacterial clumps to a single cell suspension for accurate MOI. Bacterial cell density was measured at 600 nm and the required number of bacteria was resuspended in an appropriate volume of medium (RPMI) of differentiated THP-1 at an MOI of 10 bacilli/cell to differentiated THP-1 cell. The bacteria were allowed to invade the cells for 4 hrs at 37°C in 5% CO_2_; cells were washed twice with RPMI after internalization to remove the extracellular bacteria. Amikacin (200 ng/mL) was added to infected cells which were incubated for 2 hrs, 37°C, in 5% CO_2_ to kill any extracellular bacteria. The* Mtb* infected cells were washed and harvested at specific time points after infection.

### 2.3. Preparation of Microsomes from Cultured Cells

THP-1 cells (200 × 10^6^) were grown in RPMI 1640 medium according to manufacturer protocol (Sigma Aldrich). After 24 hrs of infection, THP-1 cells were trypsinized and centrifuged at 600 g for 5 min. Supernatant was removed and the pellet washed with 10 volumes of PBS. Packed cell volume (PCV) was measured and 1x Hypotonic Extraction Buffer equivalent to 3 times the PCV was added and incubated for 20 min at 4°C to allow the cells to swell. The swollen cells were spin at 600 g for 5 min to remove the supernatant. To this, 1x Isotonic Extraction Buffer equivalent to 2 times the “new” PCV was added and samples passed 10 times through 23 gauge needle to break the cells. The homogenate thus obtained was centrifuged at 1000 g for 10 min at 4°C to separate the post nuclear supernatant that contains mitochondrial as well as microsomal fractions. It was further fractionated by centrifugation at 12000 g for 15 min at 4°C to get the post mitochondrial supernatant which is the source of microsomes.

### 2.4. Transmission Electron Microscopy

At 24 hrs after infection, the uninfected or infected THP-1 cells were used to monitor the ultrastructural changes with the assistance of transmission electron microscope (Technai 12 Biotwin TM, FEI Co., The Netherlands) [16]. Briefly, 10 × 10^6^ cells infected with either H37Rv or H37Ra and the third group that was left without infection which acts as a control were taken. Cells (~100) were taken for imaging under 80 and 100 KeV operating voltages of a transmission electron microscope (Technai 12 Biotwin TM, FEI Co., The Netherlands). Images were recorded digitally using a side-mounted 2*k* × 2*k* CCD camera (SIS, Germany).

### 2.5. SILAC Labeling and Enrichment of ER

Cells were maintained in the appropriate heavy labeled medium for 5 passages prior to differentiation with PMA and subsequent infection (Supplementary Figure  1, in Supplementary Material available online at http://dx.doi.org/10.1155/2015/270438). Lys-6 labeled media were chosen for cells to be infected with H37Ra; Lys-8 labeled media for those to be infected with H37Rv and parallel set of uninfected cells were maintained in medium containing normal lysine. At 24 hrs after infection, equal numbers (10 × 10^6^) of uninfected, H37Ra infected, and H37Rv infected cells were pooled. This cell pool was then employed for isolation of endoplasmic reticulum by using the ER Isolation kit adhering to the laid down protocol recommended by the manufacturer. From the suspension of isolated ER, protein lysate was prepared, lyophilized, and then resuspended in 100 mM ammonium bicarbonate buffer. The proteins were then subjected to denaturation and reductive alkylation prior to digestion with trypsin. Subsequently, the peptides were subjected to strong cation exchange chromatography.

### 2.6. Strong Cation Exchange (SCX)

The labeled peptide mixtures was separated by offline strong cation exchange (SCX) chromatography using an HPLC with a UV detector (1260, Binary pumps, Agilent). Briefly, labeled samples (50 *μ*g) were reconstituted in SCX running buffer (5 mM ammonium formate/30% acetonitrile) and loaded onto a Poly-LC Polysulfethyl Zorbax 300 SCX column, 5 *μ*m (2.1 mm × 150 mm, Agilent). Peptides were eluted with increasing concentrations of 5 mM ammonium formate, 30% ACN (Solvent A), to 500 mM ammonium formate, 30% ACN (Solvent B), using a gradient: 0–5 min 100% A and 0% B, 5–35 min 30% A and 70% B, 35–55 min 0% A and 100% B, 55–60 min 100% A and 0% B, and finally till 65 min on 100% A to reequilibrate the column with solvent A. Fifteen fractions were collected at a flow rate of 400 *μ*L/min according to UV trace at 220 nm. Fractions were lyophilized and were further analyzed offline on a Tempo nano-LC and spotted on MALDI plates.

### 2.7. Tempo LC MALDI Fractionation and MALDI-TOF-TOF Analysis (Bottom-Up Approach)

The peptide mixtures were analyzed by reverse-phase liquid chromatography (Tempo nano-LC from Applied Biosystems, Foster City, CA) offline coupled to AB SCIEX 5800 Proteomics Analyzer MALDI-TOF/TOF mass spectrometer (Applied Biosystems, Foster City, CA). Each fraction was reconstituted in 15 *μ*L of 1A buffer (98% water, 2% ACN and 0.1% TFA) and 12 *μ*L was picked up by auto sampler and directly loaded on to LC tempo column (Chromolith monolithic capillary, RP C18, 150 × 0.1 mm) and separated using 50 min gradient: 5% ACN to 50% ACN. The column elutes were mixed with 5 mg/mL CHCA (*α*-cyano-4 hydroxycinnamic acid) matrix in 85% acetonitrile (ACN); 0.1% TFA was spotted at a 1.5 *μ*L/min flow rate on 1364-well (44 × 31) LC-MALDI stainless steel plate. Protein identification was performed on an AB SCIEX MALDI TOFTOF 5800 Analyzer (AB SCIEX, Foster City, CA) with the mass tolerance kept at 50 ppm. For MS mode, peptide mass maps were acquired in positive reflection mode, and 800–4000 *m*/*z* mass range was used with 1500 laser shots per spectrum. The PMF peak detection criteria used include minimum signal-to-noise (*S*/*N*) of 20, local noise window width mass/charge (*m*/*z*) of 250, and minimum full-width half maximum (bins) of 1. A maximum of 30 precursors per spot with a minimum signal/noise ratio of 20 were selected for MS/MS analysis. Energy of 1 KV was used for collision-induced dissociation (CID), and 4000 acquisitions were accumulated for each MS/MS spectrum with dynamic exclusion mode of captured peptides. The peak detection criteria used were minimum *S*/*N* of 10, local noise window width (*m*/*z*) of 200, and minimum full-width half-maximum (bins) of 2.9. The interpretation for MS/MS analysis includes the exclusion of contaminant *m*/*z* peaks originating from human keratin, trypsin autodigestion, and matrix. The SILAC-labeled samples were analyzed twice on the same platform.

### 2.8. Database Search and Relative Quantification

All automatic data analysis (MS and MS/MS) and database searching were conducted against the Uniprot database (version 02-15-2014) using the ProteinPilot software (version 4.0, revision 148085, Applied Biosystems) with the Paragon method utilizing the following search parameters:* Homo sapiens *as species, trypsin as enzyme (one missed cleavage allowed), with fixed modification of methyl methanethiosulfonate (MMTS) labeled cysteine parameter enabled, SILAC-Lys6, and Lys8 labeled as sample type and the “Search Effort” parameter “Thorough ID,” which provides a broad search of various protein modifications, were chosen. The raw peptide identification results from the Paragon Algorithm (Applied Biosystems) searches were further processed by the ProGroup Algorithm (Applied Biosystems) within the ProteinPilot software before final display. Confidence threshold cut off of 95% (Unused-ProtScore > 1.3) with at least one peptide for identification and two different peptides for quantification was used. Samples from two biological replicates were analyzed together and identified a total of 133 unique human ER proteins (Supplementary Table 1).

### 2.9. Functional Characterization of the Identified Proteins

We used the UniProt Knowledgebase (UniProtKB) and Gene Ontology (GO) database information of the identified proteins to fix its ER localization, molecular function, and biological processes.

### 2.10. Extraction of Lipids by Bligh and Dyer Method

Lipids were extracted using the Bligh and dyer method [23]. Briefly, 10 × 10^6^ THP-1 cells were cultured and infected with a virulent or avirulent strains of* Mtb*. At 24 hrs after infection, THP-1 cells infected with either H37Rv or H37Ra were collected in a centrifuge tube separately. To each of these, 3.75 mL CHCl_3_ : MeOH 1 : 2 (v/v) mixture was added and vortexed. Subsequently, 1.25 mL CHCl_3_ was added and vortexed vigorously. Then again, 1.25 mL MS grade H_2_O was added and the sample vortexed for 5 min followed by centrifugation (1000 rpm, 5 min, room temperature). The lower chloroform-methanol phase was carefully recovered using the Pasteur pipette. Collected chloroform-methanol phase was further extracted once with MS grade water. Organic phase was separated and subjected to mass spectrometry through shotgun lipidomic analysis. THP-1 cells (uninfected) served as control.

### 2.11. Tandem Mass Spectrometry in Lipidomics

4000 QTRAP (ABSCIEX, Concord, Ontario, Canada) were used for phospholipids analysis by direct infusion method. Different precursor ions and neutral ions loss were assigned in our study (Supplementary Table  2). Source and compound parameters were optimized by injecting the lipids standards purchased from Avanti Polar Lipids. The source parameters were ionization spray voltage (IS) −3300, curtain gas (CUR), gas 1 (GS1), and gas 2 (GS2)—10, 10, and 5, respectively (arbitrary unit). The compound dependent parameters were declustering potential (DP), −80, entrance potential (EP), −10 V in negative polarity. Positive polarity source parameters were IS +3000, CUR, GS1, and GS2—10, 10, and 5, respectively (arbitrary unit). Compound dependent parameters were optimized as DP, 80 and EP, 80. Samples were introduced with the help of model 11 PLUS syringe pump (Harvard apparatus, Holliston, USA) at the flow rate of 10 *μ*L/min. Spectra were obtained for 100 cycles and step size is 0.1 Da with mass ranges from *m*/*z* 550 to 1000. 4000 QTRAP instrument was calibrated by the mass spectrometer standard kit provided by ABSCIEX (Foster city, CA). Phospholipids obtained from experimental sets were quantified against phospholipids standards procured from Avanti Polar Lipids (Supplementary Table  3).

### 2.12. Intracellular Ca^2+^ Measurements

Intracellular Ca^2+^ was measured with the help of Fluo-4, AM with minor modification [24]. Briefly, THP-1 cells infected with either H37Rv or H37Ra were grown. Cells on 96-well plates (15000/well/100 *μ*L) were loaded with Fluo-4, AM, (1 *μ*M, Ca^2+^ indicator) for 30 min at 37°C. Excess dye was washed; cells were resuspended with complete RPMI media and kept for 30 min to complete deesterification according to manufacturer's instructions. Then, cells were fixed with 3.7% PFA for 15 min. Images were obtained by confocal microscopy (Nikon Eclipse Ti-E laser scanning) equipped with 60x/1.4 NA PlanApochromat. DIC objective lens was excited at 488 nm and emission was recorded through emission filter set at 515/30 nm with a scanning mode format (512 × 512 pixels). Image-Pro Plus image analysis software was used for quantification. The mean fluorescence intensity/cell was determined with the help of density sum and area tools in the software. Density sum refers to the sum of intensity values of all the pixels of a counted spot. After obtaining mean fluorescence intensity/cell, the values were averaged over a minimum of 100 cells from three independent experiments. Fold increase in calcium content refers to the ratio of the averaged intensity of infected THP-1 cells versus that of uninfected THP-1 cells.

### 2.13. Western Blot Analysis

PMA differentiated THP-1 cells were infected with either H37Ra or H37Rv and another aliquot kept as uninfected. At 24 hrs after infection, the cells were homogenized in 400 *μ*L of ice-cold lysis buffer (150 mM NaCl, 50 mM HEPES pH 7.4, 1% NP-40, and 25 *μ*g/mL digitonin) and incubated on ice for 30 min. The homogenate was further centrifuged at 8500 rpm for 10 min at 4°C to get the supernatant. Supernatant from uninfected THP-1 cells was also collected as control. Protein concentrations in the supernatants were measured. Approximately 100 *μ*g of proteins from each sample was analyzed by SDS-PAGE using an 8% or a 12% resolving gel at 20 mA for 2-3 hrs. Protein bands were electroblotted onto nitrocellulose membranes (Hybond-C Extra, Amersham Biosciences). Blocking was performed for 1 hr at room temperature using Odyssey blocking buffer (Licor). For immunoblot analyses, antibodies of the following descriptions were used to probe the corresponding proteins: anti-Cyclophilin B (1 : 1000), anti-GRP94 (1 : 2000), anti-ATP2A2 (1 : 1000), anti-Liver Carboxylesterase 1 (1 : 1000), anti-AMRP (1 : 1000), anti-Calnexin (1 : 5000), and anti-HYOU1 (1 : 1000). *β*-Actin was used as a loading control. Detection involved using the goat anti-rabbit IR-dye 800 CW and goat anti-mouse IR-dye 800 CW (1 : 15000, Licor) as secondary antibodies for 90 min. Odyssey image scanner was used for scanning the images and these images were analyzed with the help of a software (Odyssey version 3.0).

### 2.14. Measurement of Caspase Activity

THP-1 cells were seeded at a density of 9 × 10^6^/6-well plate (Thermo scientific). After 24 hrs of infection, cells were scrapped and transferred to an Eppendorf tube. Caspase activity was measured as per the manufacturer's protocol (Abcam, Cambridge, MA, USA). Briefly cells were homogenized with 50 *μ*L of ice-cold cell lysis buffer and further incubated the cells on ice for 10 min. After incubation, cells were centrifuged at 10,000 g for 1 min at 4°C. Supernatants were collected and kept on ice. Protein concentrations were estimated. Approximately an aliquot of each sample (200 *μ*g) was taken. Proteins from uninfected THP-1 cells were taken as control for each experiment. Each aliquot was then solubilized in 50 *μ*L of 2X reaction buffer containing 10 mM DTT and to this 5 *μ*L of the 4 mM IETD-*p*-NA substrate (resulting in 200 *μ*M final concentration) was added and incubated for 2 hrs at 37°C. After, incubation samples were read at 405 nm in a microtiter plate reader.

## 3. Results

### 3.1. Infection with H37Ra versus H37Rv Causes Distinct Perturbations in Host Cellular Endoplasmic Reticulum (ER) Ultrastructure and Function

**Figure 1:**
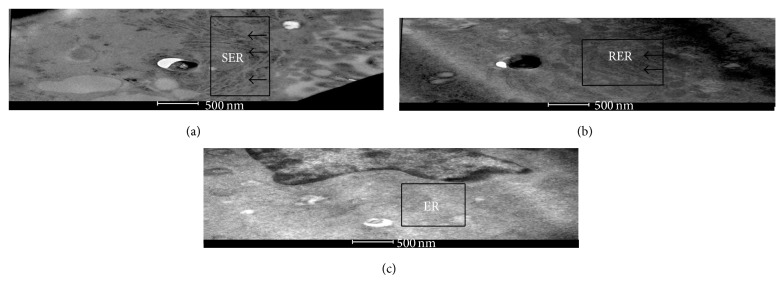
Differential changes observed in the ER morphology of THP-1 cells 24 hrs after infections with either H37Ra or H37Rv. (a) H37Ra stimulates the smooth endoplasmic reticulum as shown in electron micrograph. (b) H37Rv induces the rough endoplasmic reticulum. (c) Electron micrograph of ER (microsomes) of uninfected THP-1 cells. Endoplasmic reticulum of panels (a), (b), and (c) was denoted as SER, RER, and ER, respectively (scale bar 500 nm).

THP-1 cells (differentiated with PMA) were infected either with H37Ra or with H37Rv and were scanned to capture for any changes occurring in ultrastructure by TEM. While infection-induced alterations were evident as early as 12 hrs after infection, these changes subsequently were more prominent by 24 hrs. The functional relevance of the 24 hrs time point of observation is also supported by recent findings, as evidenced from a study of the perturbations induced in mitochondrial function [16]. We could observe prominent modifications induced in the morphology of ER on infection with avirulent H37Ra. With respect to the ER, evidence for hyperproliferation of smooth form of ER (SER) could be detected (Figure 1(a)).

In contrast to the observation made with H37Ra, examination of over 100 cells profiles by TEM showed significant morphological changes in ER in response to infection with virulent H37Rv (Figure 1(b)) which had shown the proliferation of rough form of endoplasmic reticulum (RER). This suggests that virulent* Mtb* induces an increase in protein synthesis and trafficking in the host cell soon after its entry. Previously, it has been shown that ER has the ability to modify according to the differentiation and functional state of the cells [25]. Consequently, this helps in adaptation of the ER to its various synthetic and metabolic functions [26]. For comparison, an ultrastructure of ER (microsome) of uninfected THP-1 cells was shown (Figure 1(c)).

Further to examine the adaptability of ER, we chose the proteomics and lipidomics approach to check as to whether virulence is having any impact on the host cell ER functionality. Consistent with our TEM interpretation, proteomics analysis performed on two replicate samples identified a total of 133 proteins that were known to be associated with ER (Supplementary Table  1). When a cut-off difference of at least 10-fold was applied for up- or downregulated proteins as compared to that of in uninfected cells, the relative levels of 18 proteins (Table 1) were significantly altered in response to infection and differential effects induced by H37Ra versus H37Rv were clearly evident. Similarly, lipids (phospholipids) which were differentially perturbed were enlisted in Table 2 and among them phosphatidic acid (PA), phosphatidylinositol bisphosphate (PIP2), sphingomyelin (SM), and phosphatidyl choline/phosphatidyl ethanolamine (PC/PE) were most prominent. After extensive literature survey these sets of proteins and lipids were found to be involved in specific process that alters the host cell function.

### 3.2. H37Ra Induces Accumulation of Cytosolic Ca^2+^ via Inhibiting the SERCA Activity

**Figure 2:**
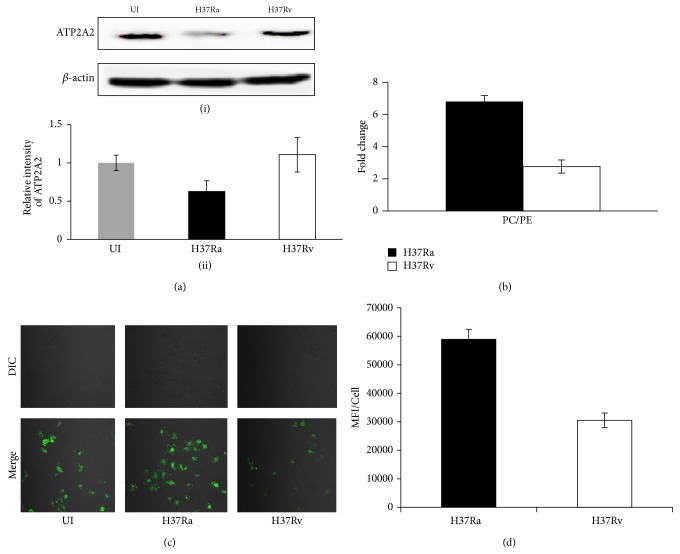
Induction in host cell cytosolic Ca^2+^ and subsequent inhibition of SERCA upon infection with H37Ra at 24 hrs. (a) (i) Representative image of western blot of ATP2A2 upon infection with H37Ra and H37Rv probed with anti-ATP2A2 primary antibody where *β*-actin was used as a loading control. Lysates from UI, H37Ra, and H37Rv from three different biological samples were used for western blot analysis. (ii) Histogram depicts the relative intensity of ATP2A2 measured in case of uninfected, H37Ra, and H37Rv, respectively. Protein was normalized to the intensity of actin and quantified. (b) Diagrammatic representation of PC/PE ratio in THP-1 cells infected with either H37Ra or H37Rv. Fold change in PC/PE ratio was 6.79 in case of H37Ra while H37Rv showed only 2.76. (c) Cytosolic Ca^2+^ levels of THP-1 cells were estimated using Confocal microscopy. Fluo-4, AM (green) was used for intracellular calcium binding and measured at 494/506 nm. (d) The mean fluorescence intensity (MFI)/cell obtained for H37Ra and H37Rv infected cells was shown. The MFI for uninfected cells was 39085 ± 580. Values represent the mean ± SE of approximately 200 cells from 3 independent experiments. White bar represents H37Rv while black bar represents H37Ra.

It is evident that SERCA has been shown to be involved in Ca^2+^ transport and maintaining the intracellular calcium levels [27]. We found that the expression levels of ATP2A2 protein (Table 1) were strongly attenuated in host cells infected with H37Ra. However, ATP2A2 is responsible for reducing the accumulation of cytosolic calcium by transferring it to ER lumen from cytosol. To examine the relative expression of ATP2A2 in THP-1 cells after an infection with either H37Ra or H37Rv, western blot analysis was employed using anti-ATP2A2 primary antibody to estimate the abundance of ER resident ATP2A2 proteins in corresponding cell lysates. The representative western blot of the ATP2A2 proteins expressed in THP-1 cells infected with either H37Ra or H37Rv was shown in Figure 2(a)(i), which is consistent with our omics results. It is evident from Figure 2(a)(ii); the relative abundance of ATP2A2 protein in THP-1 cells infected with H37Ra was markedly reduced (0.6 ± 0.14) when compared with the H37Rv infected THP-1. Further, consistency seen with the perturbation at the level of protein, our lipids, specifically PC/PE ratio (Table 2) was found to be more than sixfold in THP-1 cells infected with H37Ra compared to H37Rv (Figure 2(b)). These results indicated malfunctioning of SERCA and an insight of the accumulation of cytosolic calcium levels. So, the obvious next step was the quantitative estimation of cytosolic calcium level after an infection with either case of virulent or avirulent strain of* Mtb*. Confocal microscopy was used to monitor the intracellular levels of calcium by calcium binding Fluo-4, AM green fluorescent dye. It was observed that the cytosolic calcium level was significantly induced in the H37Ra infected THP-1 cells as shown in Figure 2(c). The quantitative estimation of the level of cytosolic calcium was obtained by calculating the mean fluorescence intensity per cell. Thus, from the relative intensity of the Fluo-4 dye which irreversibly binds to intracellular calcium it was found to be twofold higher in THP-1 cells after infection with H37Ra (Figure 2(d)).

### 3.3. H37Rv Inhibits the Apoptosis via Caspase 8 and Consequent Induction of Cholesterol Synthesis for Latent Infection

**Figure 3:**
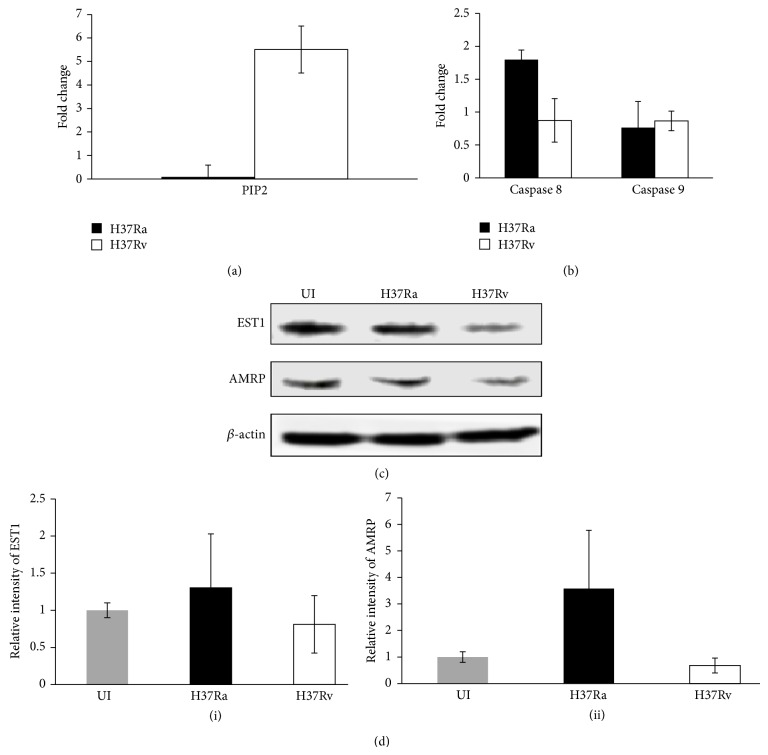
H37Rv inhibits apoptosis of THP-1 cells through caspase 8 and modulates cholesterol synthesis for latent infection. (a) Observed PIP2 levels were more than fivefold in THP-1 cells infected with H37Rv, compared to H37Ra which has direct effect on caspase 8. (b) Measured caspase activity in H37Rv infected THP-1 cells was one-half fold compared to H37Ra. Further caspase 9 activity remained unchanged. Data are representative of at least three independent experiments with similar results. (c) The cell lysates from UI, H37Ra, and H37Rv were analysed by western blot with anti-EST1 and anti-AMRP primary antibodies and *β*-actin was used as a loading control. (d) (i) Bands corresponding to EST-1 were quantified, and the intensities of each protein were normalized to the intensity of actin. (d) (ii) Similarly relative intensities of AMRP were normalized and quantified. White bar represents H37Rv while black bar represents H37Ra.

Our lipidomics results showed that the relative levels of PIP2 were found to be more than fivefold (5.5 ± 1.8) in case of THP-1 cells infected with H37Rv (Figure 3(a)). PIP2 is an established agent for the inhibition of caspase 8 and caspase 9 enzymatic activities. Next, the activities of caspase 8 and caspase 9 on infection with either H37Ra or H37Rv were monitored at 24 hrs of infection, using calorimetric assay, and we found that the expression level of caspase 8 was reduced to 50% (0.8 ± 0.3) in host cells infected with H37Rv. But surprisingly the level of expression of caspase 9 was unaltered (Figure 3(b)). Here, we can only speculate that H37Rv strain of* Mtb* might use some other pathway to counteract the host cell apoptotic challenge.

Further, our proteomics results exhibited the differential perturbations of EST1 and AMRP on the onset of infection with H37Ra and H37Rv in THP-1 cell (Table 1). To confirm this, we employed western blot technique.  Figure 3(c) shows the western blot image of the ER resident EST1 and AMRP proteins. *β*-actin was used as a loading control in each experiment. The representative western blot for visual comparison was shown in the Figure 3(d)(i). The relative intensity values of EST1 and AMRP proteins were quantitatively measured from three independent experiments. As it is evident from Figure 3(d)(ii), while the relative expression of EST1 protein was reduced significantly (0.8 ± 0.3), the relative expression of AMRP protein was down to half-fold (0.6 ± 0.2) in H37Rv infected THP-1 cells. These proteins have a potential effect on cholesterol efflux in THP-1 macrophages. These results point to the concurrent efforts of H37Rv, the virulent strain of* Mtb* to avoid the apoptosis and prepare host cells for their latent infection.

### 3.4. Effect of Perturbed ER Proteins on Host Cellular Function upon Infection with* Mtb*

It was found that several proteins (Table 1) were either directly or indirectly modulated various cellular functions which impart a possible role for host cell and bacterial cross-talk. Virulent strain of* Mtb*, that is, H37Rv, upregulated the proteins like endoplasmin (ENPL), protein disulphide-isomerase A4 (PDIA4), calnexin (CANX), peptide-prolyl cis-trans isomerase B (PPIB), glucosidase II subunit beta (GLU2B), erlin-2 (ERLN2), and cathepsin z (CTSZ). Upregulation of ENPL inhibits calpains and caspase activities by preventing an increase in calcium which leads to suppression of oxidative stress apoptosis [28]. Observed increase in PDIA4 (ER resident chaperone) levels can be associated with the termination of the classical mitochondrial apoptotic pathway [29]. Similarly, overexpression of CANX may stop the ER stress mediated apoptosis in preventing the accumulation of the nascent polypeptides [30, 31]. Reduced level of PPIB is shown to be responsible for cellular vacuolation by inducing the persistent ER stress [32], and it also activates p44/p42 MAPK and NF-*κ*B pathways which are known to reduce the proinflammatory mediators by ER stress [33]. On the other hand, GLU2B is a novel interacting protein of IP3R and enhances the activity of IP3R in vivo and enhances calcium release activity [34]. Changes in calcium concentration in the lumen of the ER play a crucial role in modulating cell sensitivity to apoptosis [35]. Thus, to counteract the higher activity of GLU2B and maintain intracellular Ca^2+^ levels, H37Rv infected macrophages upregulated the ERLN2 protein also which facilitates the ER-associated degradation of inactivated inositol 1,4,5-trisphosphate receptor (IP3R) [36]. Higher levels of CATZ (cysteine proteinases) activate Mac-1 that results in blocking of activation and proliferation of T lymphocytes and eventually modulating innate and adaptive immune response [37]. Further, highly expressed ER proteins such as CANX, PDIA4, and ERLN2 were validated by performing RT-PCR using appropriate primers (Supplementary Figure  2 and Supplementary Table  4).

Proteins such as ribophorin II (RPN2), transmembrane emp24 domain-containing protein 10 (TMED10), and phosphate disulphide isomerase 6 (PDIA6) were not detected after an infection with H37Rv. RPN2 is a transmembrane glycoprotein and an essential subunit of the N-oligosaccharyltransferase complex that catalyzes the transfer of a high mannose oligosaccharide from lipid-linked oligosaccharide donor to an asparagines residue with an Asn-X-Ser/Thr consensus motif in nascent polypeptide chains. Here we can only speculate that H37Rv employs this protein to gain entry into host macrophage cells [38]. TMED10 is a member of EMP24/GP25L/p24 family protein with GOLD domain and they bind to the COPI and COPII vesicles, thought to be involved in vesicle biosynthesis, cargo uptake, and maintaining the quality, but their actual function is still under debate [39]. However, PDIA6 is an ER resident protein and its presence exhibited the induction of unfolded protein response (UPR) [40].

Our results also indicated the downregulation of certain proteins after infection with H37Ra such as inactive rhomboid protein 2 (RHBDF2), canopy-2 (CNPY2), hypoxia upregulated protein 1 (HYOU1), and protein transport protein sec23A (SC23A). RHDF2 is a known regulator of TNF-*α* signaling [41]. It acts as a suppressor and brings about TNF-*α* induced apoptosis [42]. Since this protein is downregulated it may suggest the restoration of TNF-*α* induced apoptosis. CNPY2 and MIR-interacting saposin-like protein (Msap) act together with Mylip/idol [43] to degrade LDLR via protein ubiquitination [44]. Since, in our findings, CNPY2 is downregulated in H37Ra infected macrophages it may upregulate the Mylip/idol signaling by loss of gain function which in turn downregulates the LDLR thereby marginally increasing the cholesterol level. The marginal suppression of HYOU1 was observed after an infection with H37Ra of THP-1 cell. This is hypoxia-inducible stress protein, demonstrated to have a cytoprotective role, and its overexpression assisted the macrophage survival [45]. Thus again by loss of gain function the H37Ra infected macrophages may undergo apoptosis readily. Lastly, SC23A that is important for the transportation of newly synthesized secretory proteins from the endoplasmic reticulum to the golgi [46] is also downregulated suggesting the activation of unfolded protein response.

## 4. Discussion

Basically an intracellular pathogen,* Mtb*, is amply equipped with successful strategies to overcome the host's immune response and the rising rates of tuberculosis cases are a proof of its success as a pathogen. It is plausible that the mycobacteria may integrate several cellular mechanisms for their survival. That averting of host macrophage apoptosis plays a key survival strategy of* Mtb* and it is shown from previous studies that infection with susceptible strains of* Mtb* such as H37Ra or* M. bovis BCG* rapidly amplified the macrophage apoptosis [47]. On the other hand virulent strains are successful in stalling the apoptotic response. Furthermore, at some later phase, they favor to trigger necrosis so as to allow bacterial spread into the new uninfected host cells [48]. An unanswered question that still continues, however, is the nature of perturbations that virulent* Mtb* hold on the host cell, in order to offset the early activation of apoptotic pathways, but little is revealed on whether* Mtb* also perturbs the ER for the initiation of this process at a very early stage of infection.

We, therefore, undertook the present study to explore the answer of this question. A comprehensive investigation of this issue clearly needs a thorough and in-depth analysis. Therefore, the current work was constructed more as an illustrative investigation, to provide whether the differential THP-1 cellular perturbations to either H37Ra or H37Rv infection could be drawn at the level of early modulations in ER function.

Thus, ER in cells infected with either H37Ra or H37Rv showed distinct alterations in ultrastructure. Of particular interest though was the fact that these distinct outcomes were indeed marked soon after infection, at the level of architectural and functional perturbations in the ER of the host cell. In addition, H37Ra induced the transformation of ER to SER and in reverse H37Rv promoted the induction of RER. These results, therefore, suggested investigating whether the observed variations could be explained through regulation in ER proteome and lipidome.

**Figure 4:**
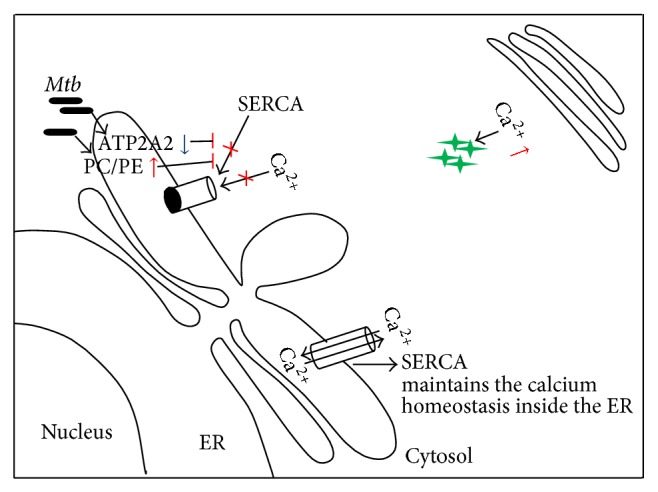
Schematic representation of ER perturbations upon H37Ra infection of THP-1 cell. ATP2A2 was downregulated in H37Ra infected cells and this inhibits SERCA activity. Simultaneous increase in PC/PE ratio also blocks SERCA activity, with the consequent increase in cytosolic calcium. This, in turn, likely promotes phagolysosomal fusion and consequent elimination of the mycobacteria. However, SERCA in normal condition is responsible for the maintenance of calcium homeostasis. Blue arrow indicates the downregulation while red arrow represents the upregulation.

Although our present endoplasmic reticulum proteomic study was somewhat limited in scope the results of our quantitative analysis, nonetheless, yield a ready rationalization for the distinct consequences of H37Ra versus H37Rv infection. Thus, for example, the increased cytosolic calcium level by endoplasmic reticulum in H37Ra infected cells was observed, at the functional level, by the specific reduction of ATP2A2 in these cells. In addition, these cells affected the level of PC/PE ratio. As indicated, changes in the PC/PE balance in a cellular setting can significantly perturb SERCA function [49]. The observed modulation in SERCA function eventually facilitates the phagosome-lysosome fusion [50]. Further, the perturbation in THP-1 cells after an infection with H37Ra was diagrammatically depicted in Figure 4.

Notably, our results also provided a preliminary insight into the mechanisms entailed in the H37Rv-dependent suppression of cellular apoptosis. Earlier studies suggest a very distinct cell death mode induced by virulent strains of* Mtb* from the one induced by avirulent one. Similarly, from our ultrastructural observations, it is quite clear that the ultrastructural features of ER in case of H37Rv infected cells are very different from those observed in H37Ra infected cell. Further to support our ultrastructural findings some of the proteins like PPIB, ENPL, CANX, PDIA4, and PDIA6 regulate cell death directly or indirectly. These proteins have antiapoptotic activity which is markedly upregulated and would at least partly be responsible for this consequence. Such an interpretation is backed by the fact that these proteins were not induced after an infection with H37Ra. On the contrary, significantly induced level of the SM supports the ultrastructural state of ER seen in cells infected with H37Ra. SM has been implicated to function as an inducer of apoptosis by increasing the expression of TNF-*α* and caspase 3 mRNA level [51]. In accordance with the observed ER morphology, it is tempting to speculate that cells upon H37Ra infection self-induce destabilization of ER eventually leading to death.

**Figure 5:**
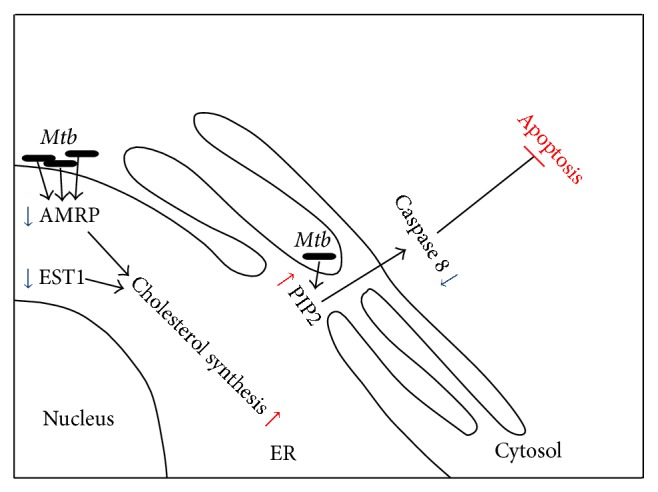
Graphical representation of ER perturbation upon H37Rv infection of THP-1 cell. Decrease in AMRP and EST1 proteins is suggestive of cholesterol accumulation. This increase in cholesterol assists in* Mtb* persistence. Along with cholesterol accumulation, H37Rv also induces the expression level of PIP2 which in turn is a direct inhibitor of caspase 8 leading to the inhibition of cellular apoptosis. Blue arrow is for downregulation and red arrow shows upregulation.

The host lipids metabolic pathway related to* Mtb* virulence specific distinction is also of particular interest. Interestingly, we have recently shown that only virulent* Mtb* strains can induce LBs in the THP-1 cell, and that both H37Ra and* M. smegmatis* strains lack this capability [52]. In our present study, expression of LRPAP1 protein was downregulated in cells infected with H37Rv which helps in folding the LDL receptor family proteins. Apart from this protein, EST1 was also strongly suppressed in H37Rv infected cells. Since EST1 is responsible for the total cholesterol ester hydrolytic activity [53], it is likely that macrophages maintain the cholesterol in its esterified form in the foam cells and the finding of EST1 that is an endoplasmic reticulum protein would certainly highlight the role of endoplasmic reticulum in making the foamy macrophage. In addition to these proteins, our lipid results indicated the upregulation of PIP2 in cells infected with H37Rv. Previously, PIP2 has been demonstrated to be an inhibitor of caspase 8 and caspase 9 [54]. But surprisingly, our result indicated the reduction of only caspase 8 but not caspase 9 which remains unperturbed. Thus again, Figure 5 is a representation of modulations brought in human macrophages by H37Rv.

It is therefore evident from the above results that an analysis of the endoplasmic reticulum proteome and lipidome indeed provided some glimpse into the molecular mechanisms between the host and the* Mtb* pathogen. Importantly, our approach of correlating the H37Ra versus H37Rv infection also proved useful as it yielded an insight into the mechanisms by which virulent* Mtb* modulates host cellular consequences. However, indeed still of a preliminary in nature, our results also highlight the combined nature of the effects that H37Rv exercises on the host macrophage. Thus, the early inhibition of apoptosis and the support in activation of LB, collectively ensure the substantial persistence of the virulent bacilli in the macrophages. Therefore, this study features utility of performing a more comprehensive examination of the* Mtb*-induced changes in the host cell endoplasmic reticulum proteome. Significantly, a time-course functional analysis for the entire duration of infection can be expected to yield new and vital information on the mechanisms that equips the adaptive immune response of virulent* Mtb* within the macrophage.

## 5. Conclusions

In conclusion, interrogation of ultrastructural changes in ER observed through TEM and then ER proteome and lipidome of* Mtb* infected macrophages provided a preliminary glimpse of the underlying changes that occur at the protein and lipids level, thereby also rationalizing the detected alterations in functional properties. The studies described here yield new insights into the host-pathogen interplay that occurs in* Mtb* infected macrophages. Importantly, these studies provide an integrated perspective on the strategies adopted by the virulent pathogen, in order to successfully adapt within the hostile intracellular milieu of the macrophage. More detailed studies in this direction are likely to further enhance our understanding of these processes and can then aid in the development of novel approaches for the treatment of TB.

## Supplementary Material

Supplementary materials contain four tables and two figures for additional information. Table S1 contains the list of 133 ER resident proteins that were identified using SILAC strategy employing Lys-6 and Lys-8 as the heavy labels. Table S2 shows the optimized collision energy (CE) used to generate the fragmentation of head groups of 8 phospholipids under study. Table S3 provides the quantitative estimation of 8 phospholipids from the peak area compared against the corresponding phospholipids standards peak area. Table S4 presents the information of the sequences and product size of the primers corresponding to CANX, PDIA4 and ERLN2. Figure S1 shows the schematic representation of the SILAC labelling method used for the quantitative proteomics approach and lastly figure S2 depicts the observed upregulation of the host cell CANX, PDIA4 and ERLN2 upon infection with H37Rv as against H37Ra.

## Figures and Tables

**Table 1:** Representation of the list of 18 ER proteins in THP-1 cells infected with either H37Ra or H37Rv.

	Accession number	Protein identity	Name	H37Ra	H37Rv	Functions
1	P16615	ATP2A2	Sarcoplasmic/endoplasmic reticulum calcium ATPase 2	0.0121	0.3839	Calcium homeostasis

2	P23141	EST1 or CES1	Liver carboxylesterase 1	1.6828	ND	Cholesterol homeostasis

3	P30533	AMRP	Alpha-2-macroglobulin receptor-associated protein	2.0374	ND	Cholesterol homeostasis

4	P14625	ENPL	Endoplasmin	0.9387	15.9431	Calcium homeostasis

5	P13667	PDIA4	Protein disulfide-isomerase A4	1.3674	21.4636	Termination of classical mitochondrial apoptotic pathway

6	P27824	CANX	Calnexin	1.2835	27.1939	Stops ER stress mediated apoptosis

7	P23284	PPIB	Peptidyl-prolyl cis-trans isomerase B	0.8023	10.6105	NF-*κ*B pathway

8	P14314	GLU2B	Glucosidase 2 subunit beta	1.1434	16.7416	Novel interactor of IP3R

9	O94905	ERLN2	Erlin-2	1.2078	21.7272	ER associated degradation of activated IP3R

10	Q9UBR2	CTSZ	Cathepsin Z	1.0487	10.9698	Diminished activation and proliferation of T-lymphocytes

11	P04844	RPN2	Dolichyl-diphosphooligosaccharide	0.9903	ND	Unknown

12	P49755	TMED10	Transmembrane emp24 domain-containing protein 10	1.212	ND	Vesicle biogenesis, Cargo uptake

13	Q15084	PDIA6	Protein disulfide-isomerase A6	1.483	ND	Unfold protein response

14	Q6PJF5	RHBDF2	Inactive rhomboid protein 2	0.0121	0.3839	TNF-*α* signaling

15	Q9Y2B0	CNPY2	Protein canopy homolog 2	0.0121	0.3839	Cholesterol homeostasis

16	Q9Y4L1	HYOU1	Hypoxia upregulated protein 1	0.1393	0.3839	Cytoprotective role

17	Q15436	SC23A	Protein transport protein Sec23A	0.0121	0.3839	Transportation of proteins from ER to golgi

18	O75844	FACE1	CAAX prenyl protease 1 homolog	0.0121	0.3839	Maintaining normal cell functions

Up- or downregulated proteins were selected on the basis of a cut-off of at least a 10-fold difference in the level as compared to that in uninfected cells. ND indicates protein not detected.

**Table 2:** List of phospholipids in THP-1 cells infected with either H37Ra or H37Rv by shotgun lipidomics approach.

Name of lipids	H37Ra	H37Rv
PG	0.41	0.63
PA	0.30	0.78
PI	0.11	0.18
PIP2	0.89	5.6
PE	0.28	0.80
PS	0.74	0.69
PC	1.29	2.22
SM	5.21	2.14
PC/PE	6.79	2.76

Phosphatidyl glycerol (PG), phosphatidic acid (PA), phosphatidyl inositol (PI), phosphatidyl inositol bisphosphate (PIP2), phosphatidyl ethanolamine (PE), phosphatidyl serine (PS), phosphatidyl choline (PC), and sphingomyelin (SM) were identified confidently. Among them, PC/PE ratio, PIP2, and SM were shown to be differentially altered. Data is representative of the three biological repeats after normalization with control sample.

## Abbreviations

RPMI 1640: Roswell Park Memorial Institute 1640
ACN: Acetonitrile
*Mtb*: * Mycobacterium tuberculosis*
CUR: Curtain gas
DP: Declustering potential
EP: Entrance potential
CE: Collision energy
Da: Dalton
Lys-6: Lysine-6
Lys-8: Lysine-8
PFA: Paraformaldehyde
NP-40: (Octylphenoxy) polyethoxyethanol
DTT: Dithiothreitol
IETD-*p*-NA: Ile-Glu-Thr-Asp conjugated to* p*-nitroaniline
MOI: Multiplicity of infection
TEM: Transmission electron microscopy
MALDI-TOF/TOF: Matrix-assisted laser desorption/ionization time-of-flight/time-of-flight
2D-LC/MS/MS: 2-dimensional-liquid chromatography tandem mass spectrometry
SCX: Strong cation exchange
SILAC: Stable isotope labeling by amino acids in cell culture
ER: Endoplasmic reticulum.
