# Supplementary material for: Quantitative Proteomics and Lipidomics Analysis of Endoplasmic Reticulum of Macrophage Infected with Mycobacterium tuberculosis
Source: Int J Proteomics. 2015 Feb 16;2015:270438. doi: 10.1155/2015/270438 (PMC4345262; doi:10.1155/2015/270438)
Supplement: Supplementary file 1 — Supplementary materials contain four tables and two figures for additional information. Table S1 contains the list of 133 ER resident proteins that were identified using SILAC strategy employing Lys-6 and Lys-8 as the heavy labels. Table S2 shows the optimized collision energy (CE) used to generate the fragmentation of head groups of 8 phospholipids under study. Table S3 provides the quantitative estimation of 8 phospholipids from the peak area compared against the corresponding phospholipids standards peak area. Table S4 presents the information of the sequences and product size of the primers corresponding to CANX, PDIA4 and ERLN2. Figure S1 shows the schematic representation of the SILAC labelling method used for the quantitative proteomics approach and lastly figure S2 depicts the observed upregulation of the host cell CANX, PDIA4 and ERLN2 upon infection with H37Rv as against H37Ra. [file 270438.f1.pdf]

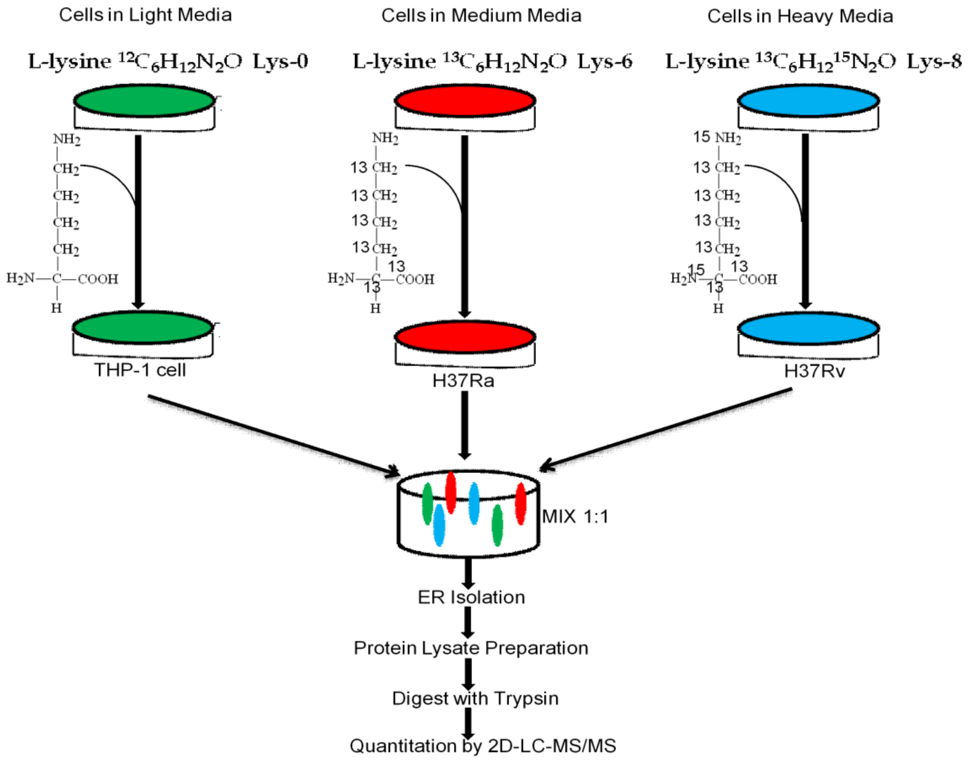

SUPPLEMENTARY FIGURE 1

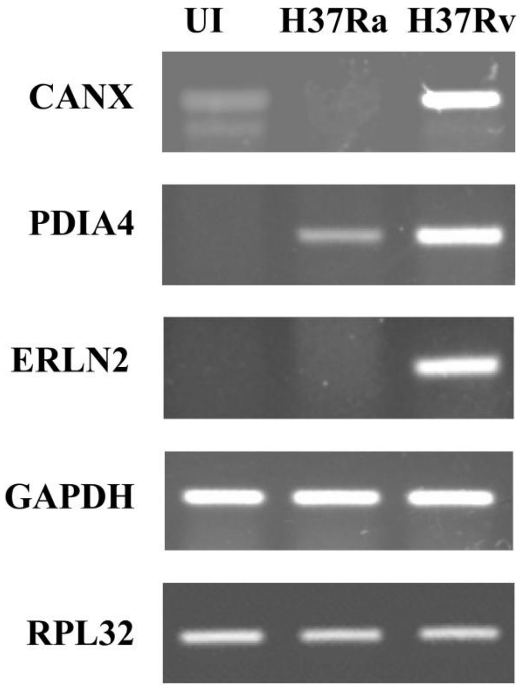

SUPPLEMENTARY FIGURE-2

S.No	Unused ProtScore	Coverage %	Gene ID_Accession Number
1	49.3	56.1	P11021
2	35.24	46.1	P07237
3	32.18	36.7	P14625
4	29.92	49.5	P30101
5	29.49	61.9	P27797
6	28.34	53.6	P04222
7	25.56	31.6	Q14697
8	22.75	37.6	P04843
9	19.54	50.4	P01892
10	15.38	30.2	P27824
11	14.53	36.8	Q15084
12	12.75	19.7	Q9Y4L1
13	12.55	21.6	P04844
14	12.28	25.9	P13667
15	12.23	38.4	P23284
16	12.02	20.4	Q02809
17	11.66	35.1	P27635
18	10.79	26.6	P23141
19	10.32	27.1	P14314
20	8.8	28	Q03518
21	8.72	24.1	Q9UBR2
22	8.46	27.3	Q13501
23	8.32	30.8	O15533
24	7.95	22.8	O60506
25	6.77	37.6	O00560
26	6.2	7.6	Q9NQC3
27	5.62	15.2	P16615
28	5.38	29.3	O75396
29	5.34	27.5	Q99541
30	5.23	23.1	Q32P28
31	5.22	17.1	P49768
32	5.18	15.5	P08240
33	5.14	19.1	Q8NBJ5
34	5.06	14.5	P49257
35	4.94	15	Q8TCJ2
36	4.92	17.4	Q6PJF5
37	4.88	18	P39656
38	4.88	24.1	Q9BS26
39	4.77	16	P20591
40	4.74	18.2	O15260
41	4.48	11.2	Q8IV08
42	4.36	23.2	P13674
43	4.34	27.4	P27695
44	4.27	30	P30533
45	4.24	18.5	Q13724
46	4	28.1	P09601
47	4	27.4	P51151
48	3.94	21.4	Q12907
49	3.94	20.7	Q96CS3

50	3.82	13.2	Q9UBV2
51	3.8	13.2	Q9BUN8
52	3.65	34.9	Q9UBS4
53	3.51	13.3	P43307
54	3.51	27.1	P60468
55	3.51	9.6	Q14534
56	3.51	21	Q9BTV4
57	3.48	40.1	Q9Y2B0
58	3.35	23.6	O75340
59	3.27	18.3	O75844
60	3.22	25.4	Q15293
61	3.15	9.5	P46977
62	3.07	20.7	P16435
63	2.88	23.6	P61019
64	2.88	24.5	Q6RW13
65	2.81	19.8	Q9Y385
66	2.63	13.3	O94905
67	2.63	12.4	Q15005
68	2.56	25.4	O43852
69	2.47	11.5	O60568
70	2.46	10.2	Q9NZ08
71	2.41	10.3	Q15437
72	2.36	28	P50454
73	2.31	12.8	O43865
74	2.26	8.1	Q9NZ01
75	2.22	23.9	Q96HY6
76	2.21	12.9	Q14257
77	2.15	8.7	Q7Z2K6
78	2.1	17.4	Q969V3
79	2.09	18.3	Q01105
80	2.08	11.2	Q9H1C4
81	2.05	25.6	Q86UE4
82	2.04	14.7	Q14573
83	2.03	37.4	P49755
84	2.02	20.4	Q15363
85	2.02	7.9	Q96HE7
86	2	8.6	Q14165
87	2	11.9	Q3ZAQ7
88	2	7.9	Q6DD88
89	2	33.3	Q8WUD1
90	2	16.3	Q96AG4
91	2	10.2	Q9UL25
92	2	11.9	Q9UNL2
93	1.86	5.6	Q8TCT9
94	1.71	10	O95479
95	1.59	6.1	Q92643
96	1.51	7.4	A5PLL7
97	1.43	12.7	Q9NYU2
98	1.38	10.1	Q15436
99	1.34	5	O95197

100	1.24	10.7	O95433
101	1.23	19.8	Q9P2E9
102	1.16	7.8	P61619
103	1.15	14.8	Q9BVC6
104	1.12	8.6	P37268
105	1.09	6.5	P53992
106	1.07	10.4	Q8N5M9
107	1.05	14.6	O94874
108	1.05	13	Q9Y3B3
109	1.02	10.8	Q13438
110	1	10.2	P23219
111	0.98	10.7	Q9H3N1
112	0.98	9.6	Q8NF37
113	0.96	4.7	P49810
114	0.81	26.4	Q9BVK6
115	0.78	4	P55735
116	0.76	5.2	Q96DZ1
117	0.73	8.8	Q9BVG9
118	0.67	18	Q13190
119	0.63	20.5	O00264
120	0.61	12.1	Q12797
121	0.6	5.1	P48651
122	0.51	7.8	Q14643
123	0.51	14.1	Q96S52
124	0.49	4.9	P48723
125	0.47	7.6	Q9NQW1
126	0.44	2.9	Q6PIU2
127	0.4	14.9	Q9P035
128	0.39	17.3	Q9Y5M8
129	0.35	19	P67812
130	0.34	22.2	Q9HCN8
131	0.3	10.4	Q8N2G8
132	0.29	17.8	Q9UNK0
133	0.24	23.9	Q9P2X0

Supplementary Table-1
ND- Not detected
ER- Endoplasmic reticulum

Gene Name
GRP78
PDIA1
ENPL
PDIA3
CALR
1C03
GANAB
RPN1
1A02
CALX
PDIA6
HYOU1
RPN2
PDIA4
PPIB
PLOD1
RL10
EST1
GLU2B
TAP1
CATZ
SQSTM
TPSN
HNRPQ
SDCB1
RTN4
AT2A2
SC22B
PLIN2
P3H1
PSN1
SRPR
GT251
LMAN1
STT3B
RHDF2
OST48
ERP44
MX1
SURF4
PLD3
P4HA1
APEX1
AMRP
MOGS
HMOX1
RAB9A
LMAN2
FAF2

SE1L1
DERL1
DJB11
SSRA
SC61B
ERG1
TMM43
CNPY2
PDCD6
FACE1
RCN1
STT3A
NCPR
RAB2A
ATRAP
UB2J1
ERLN2
SPCS2
CALU
PLOD3
ERAP1
SC23B
SERPH
SAHH2
TECR
DDRGK
RCN2
ERMP1
NCLN
SET
UN93B
LYRIC
ITPR3
TMEDA
TMED2
ERO1A
MLEC
VMA21
ATLA3
RAB2B
LRC59
RAB21
SSRG
HM13
G6PE
GPI8
TM189
UGGG1
SC23A
RTN3

AHSA1
RRBP1
S61A1
TM109
FDFT
SC24C
JAGN1
UFL1
TMED7
OS9
PGH1
TMX1
PCAT1
PSN2
TMED9
SEC13
ERLEC
PTSS2
STX5
PGRC1
ASPH
PTSS1
ITPR1
PIGS
HSP13
SC31B
NCEH1
HACD3
SRPRB
SC11A
SDF2L
GHDC
STX8
DPM3

Protein ID
78 kDa glucose-regulated protein OS=Homo sapiens GN=HSPA5 PE=1 SV=2
Protein disulfide-isomerase OS=Homo sapiens GN=P4HB PE=1 SV=3
Endoplasmin OS=Homo sapiens GN=HSP90B1 PE=1 SV=1
Protein disulfide-isomerase A3 OS=Homo sapiens GN=PDIA3 PE=1 SV=4
Calreticulin OS=Homo sapiens GN=CALR PE=1 SV=1
HLA class I histocompatibility antigen, Cw-3 alpha chain OS=Homo sapiens GN=HLA-C PE=1 SV=2
Neutral alpha-glucosidase AB OS=Homo sapiens GN=GANAB PE=1 SV=3
Dolichyl-diphosphooligosaccharide--protein glycosyltransferase subunit 1 OS=Homo sapiens GN=RPN1 PE=1
HLA class I histocompatibility antigen, A-2 alpha chain OS=Homo sapiens GN=HLA-A PE=1 SV=1
Calnexin OS=Homo sapiens GN=CANX PE=1 SV=2
Protein disulfide-isomerase A6 OS=Homo sapiens GN=PDIA6 PE=1 SV=1
Hypoxia up-regulated protein 1 OS=Homo sapiens GN=HYOU1 PE=1 SV=1
Dolichyl-diphosphooligosaccharide--protein glycosyltransferase subunit 2 OS=Homo sapiens GN=RPN2 PE=1
Protein disulfide-isomerase A4 OS=Homo sapiens GN=PDIA4 PE=1 SV=2
Peptidyl-prolyl cis-trans isomerase B OS=Homo sapiens GN=PPIB PE=1 SV=2
Procollagen-lysine,2-oxoglutarate 5-dioxygenase 1 OS=Homo sapiens GN=PLOD1 PE=1 SV=2
60S ribosomal protein L10 OS=Homo sapiens GN=RPL10 PE=1 SV=4
Liver carboxylesterase 1 OS=Homo sapiens GN=CES1 PE=1 SV=2
Glucosidase 2 subunit beta OS=Homo sapiens GN=PRKCSH PE=1 SV=2
Antigen peptide transporter 1 OS=Homo sapiens GN=TAP1 PE=1 SV=2
Cathepsin Z OS=Homo sapiens GN=CTSZ PE=1 SV=1
Sequestosome-1 OS=Homo sapiens GN=SQSTM1 PE=1 SV=1
Tapasin OS=Homo sapiens GN=TAPBP PE=1 SV=1
Heterogeneous nuclear ribonucleoprotein Q OS=Homo sapiens GN=SYNCRIP PE=1 SV=2
Syntenin-1 OS=Homo sapiens GN=SDCBP PE=1 SV=1
Reticulon-4 OS=Homo sapiens GN=RTN4 PE=1 SV=2
Sarcoplasmic/endoplasmic reticulum calcium ATPase 2 OS=Homo sapiens GN=ATP2A2 PE=1 SV=1
Vesicle-trafficking protein SEC22b OS=Homo sapiens GN=SEC22B PE=1 SV=4
Perilipin-2 OS=Homo sapiens GN=PLIN2 PE=1 SV=2
Prolyl 3-hydroxylase 1 OS=Homo sapiens GN=LEPRE1 PE=1 SV=2
Presenilin-1 OS=Homo sapiens GN=PSEN1 PE=1 SV=1
Signal recognition particle receptor subunit alpha OS=Homo sapiens GN=SRPR PE=1 SV=2
Procollagen galactosyltransferase 1 OS=Homo sapiens GN=GLT25D1 PE=1 SV=1
Protein ERGIC-53 OS=Homo sapiens GN=LMAN1 PE=1 SV=2
Dolichyl-diphosphooligosaccharide--protein glycosyltransferase subunit STT3B OS=Homo sapiens GN=STT3B
Inactive rhomboid protein 2 OS=Homo sapiens GN=RHBDF2 PE=1 SV=2
Dolichyl-diphosphooligosaccharide--protein glycosyltransferase 48 kDa subunit OS=Homo sapiens GN=DDOS
Endoplasmic reticulum resident protein 44 OS=Homo sapiens GN=ERP44 PE=1 SV=1
Interferon-induced GTP-binding protein Mx1 OS=Homo sapiens GN=MX1 PE=1 SV=4
Surfeit locus protein 4 OS=Homo sapiens GN=SURF4 PE=1 SV=3
Phospholipase D3 OS=Homo sapiens GN=PLD3 PE=1 SV=1
Prolyl 4-hydroxylase subunit alpha-1 OS=Homo sapiens GN=P4HA1 PE=1 SV=2
DNA-(apurinic or apyrimidinic site) lyase OS=Homo sapiens GN=APEX1 PE=1 SV=2
Alpha-2-macroglobulin receptor-associated protein OS=Homo sapiens GN=LRPAP1 PE=1 SV=1
Mannosyl-oligosaccharide glucosidase OS=Homo sapiens GN=MOGS PE=1 SV=5
Heme oxygenase 1 OS=Homo sapiens GN=HMOX1 PE=1 SV=1
Ras-related protein Rab-9A OS=Homo sapiens GN=RAB9A PE=1 SV=1
Vesicular integral-membrane protein VIP36 OS=Homo sapiens GN=LMAN2 PE=1 SV=1
FAS-associated factor 2 OS=Homo sapiens GN=FAF2 PE=1 SV=2

Protein sel-1 homolog 1 OS=Homo sapiens GN=SEL1L PE=1 SV=3
Derlin-1 OS=Homo sapiens GN=DERL1 PE=1 SV=1
DnaJ homolog subfamily B member 11 OS=Homo sapiens GN=DNAJB11 PE=1 SV=1
Translocon-associated protein subunit alpha OS=Homo sapiens GN=SSR1 PE=1 SV=3
Protein transport protein Sec61 subunit beta OS=Homo sapiens GN=SEC61B PE=1 SV=2
Squalene monooxygenase OS=Homo sapiens GN=SQLE PE=2 SV=3
Transmembrane protein 43 OS=Homo sapiens GN=TMEM43 PE=1 SV=1
Protein canopy homolog 2 OS=Homo sapiens GN=CNPY2 PE=1 SV=1
Programmed cell death protein 6 OS=Homo sapiens GN=PDCD6 PE=1 SV=1
CAAX prenyl protease 1 homolog OS=Homo sapiens GN=ZMPSTE24 PE=1 SV=2
Reticulocalbin-1 OS=Homo sapiens GN=RCN1 PE=1 SV=1
Dolichyl-diphosphooligosaccharide--protein glycosyltransferase subunit STT3A OS=Homo sapiens GN=STT3A
NADPH--cytochrome P450 reductase OS=Homo sapiens GN=POR PE=1 SV=2
Ras-related protein Rab-2A OS=Homo sapiens GN=RAB2A PE=1 SV=1
Type-1 angiotensin II receptor-associated protein OS=Homo sapiens GN=AGTRAP PE=1 SV=1
Ubiquitin-conjugating enzyme E2 J1 OS=Homo sapiens GN=UBE2J1 PE=1 SV=2
Erlin-2 OS=Homo sapiens GN=ERLIN2 PE=1 SV=1
Signal peptidase complex subunit 2 OS=Homo sapiens GN=SPCS2 PE=1 SV=3
Calumenin OS=Homo sapiens GN=CALU PE=1 SV=2
Procollagen-lysine,2-oxoglutarate 5-dioxygenase 3 OS=Homo sapiens GN=PLOD3 PE=1 SV=1
Endoplasmic reticulum aminopeptidase 1 OS=Homo sapiens GN=ERAP1 PE=1 SV=3
Protein transport protein Sec23B OS=Homo sapiens GN=SEC23B PE=1 SV=2
Serpin H1 OS=Homo sapiens GN=SERPINH1 PE=1 SV=2
Putative adenosylhomocysteinase 2 OS=Homo sapiens GN=AHCYL1 PE=1 SV=2
Trans-2,3-enoyl-CoA reductase OS=Homo sapiens GN=TECR PE=1 SV=1
DDRGK domain-containing protein 1 OS=Homo sapiens GN=DDRGK1 PE=1 SV=2
Reticulocalbin-2 OS=Homo sapiens GN=RCN2 PE=1 SV=1
Endoplasmic reticulum metallopeptidase 1 OS=Homo sapiens GN=ERMP1 PE=1 SV=2
Nicalin OS=Homo sapiens GN=NCLN PE=1 SV=2
Protein SET OS=Homo sapiens GN=SET PE=1 SV=3
Protein unc-93 homolog B1 OS=Homo sapiens GN=UNC93B1 PE=1 SV=2
Protein LYRIC OS=Homo sapiens GN=MTDH PE=1 SV=2
Inositol 1,4,5-trisphosphate receptor type 3 OS=Homo sapiens GN=ITPR3 PE=1 SV=2
Transmembrane emp24 domain-containing protein 10 OS=Homo sapiens GN=TMED10 PE=1 SV=2
Transmembrane emp24 domain-containing protein 2 OS=Homo sapiens GN=TMED2 PE=1 SV=1
ERO1-like protein alpha OS=Homo sapiens GN=ERO1L PE=1 SV=2
Malectin OS=Homo sapiens GN=MLEC PE=1 SV=1
Vacuolar ATPase assembly integral membrane protein VMA21 OS=Homo sapiens GN=VMA21 PE=1 SV=1
Atlastin-3 OS=Homo sapiens GN=ATL3 PE=1 SV=1
Ras-related protein Rab-2B OS=Homo sapiens GN=RAB2B PE=1 SV=1
Leucine-rich repeat-containing protein 59 OS=Homo sapiens GN=LRRC59 PE=1 SV=1
Ras-related protein Rab-21 OS=Homo sapiens GN=RAB21 PE=1 SV=3
Translocon-associated protein subunit gamma OS=Homo sapiens GN=SSR3 PE=1 SV=1
Minor histocompatibility antigen H13 OS=Homo sapiens GN=HM13 PE=1 SV=1
GDH/6PGL endoplasmic bifunctional protein OS=Homo sapiens GN=H6PD PE=1 SV=2
GPI-anchor transamidase OS=Homo sapiens GN=PIGK PE=1 SV=2
Transmembrane protein 189 OS=Homo sapiens GN=TMEM189 PE=2 SV=3
UDP-glucose:glycoprotein glucosyltransferase 1 OS=Homo sapiens GN=UGGT1 PE=1 SV=3
Protein transport protein Sec23A OS=Homo sapiens GN=SEC23A PE=1 SV=2
Reticulon-3 OS=Homo sapiens GN=RTN3 PE=1 SV=2

Activator of 90 kDa heat shock protein ATPase homolog 1 OS=Homo sapiens GN=AHSA1 PE=1 SV=1
Ribosome-binding protein 1 OS=Homo sapiens GN=RRBP1 PE=1 SV=4
Protein transport protein Sec61 subunit alpha isoform 1 OS=Homo sapiens GN=SEC61A1 PE=1 SV=2
Transmembrane protein 109 OS=Homo sapiens GN=TMEM109 PE=1 SV=1
Squalene synthase OS=Homo sapiens GN=FDFT1 PE=1 SV=1
Protein transport protein Sec24C OS=Homo sapiens GN=SEC24C PE=1 SV=3
Protein jagunal homolog 1 OS=Homo sapiens GN=JAGN1 PE=1 SV=1
E3 UFM1-protein ligase 1 OS=Homo sapiens GN=UFL1 PE=1 SV=2
Transmembrane emp24 domain-containing protein 7 OS=Homo sapiens GN=TMED7 PE=1 SV=2
Protein OS-9 OS=Homo sapiens GN=OS9 PE=1 SV=1
Prostaglandin G/H synthase 1 OS=Homo sapiens GN=PTGS1 PE=1 SV=2
Thioredoxin-related transmembrane protein 1 OS=Homo sapiens GN=TMX1 PE=1 SV=1
Lysophosphatidylcholine acyltransferase 1 OS=Homo sapiens GN=LPCAT1 PE=1 SV=2
Presenilin-2 OS=Homo sapiens GN=PSEN2 PE=1 SV=1
Transmembrane emp24 domain-containing protein 9 OS=Homo sapiens GN=TMED9 PE=1 SV=2
Protein SEC13 homolog OS=Homo sapiens GN=SEC13 PE=1 SV=3
Endoplasmic reticulum lectin 1 OS=Homo sapiens GN=ERLEC1 PE=1 SV=1
Phosphatidylserine synthase 2 OS=Homo sapiens GN=PTDSS2 PE=1 SV=1
Syntaxin-5 OS=Homo sapiens GN=STX5 PE=1 SV=2
Membrane-associated progesterone receptor component 1 OS=Homo sapiens GN=PGRMC1 PE=1 SV=3
Aspartyl/asparaginyl beta-hydroxylase OS=Homo sapiens GN=ASPH PE=1 SV=3
Phosphatidylserine synthase 1 OS=Homo sapiens GN=PTDSS1 PE=1 SV=1
Inositol 1,4,5-trisphosphate receptor type 1 OS=Homo sapiens GN=ITPR1 PE=1 SV=2
GPI transamidase component PIG-S OS=Homo sapiens GN=PIGS PE=1 SV=3
Heat shock 70 kDa protein 13 OS=Homo sapiens GN=HSPA13 PE=1 SV=1
Protein transport protein Sec31B OS=Homo sapiens GN=SEC31B PE=1 SV=1
Neutral cholesterol ester hydrolase 1 OS=Homo sapiens GN=NCEH1 PE=1 SV=3
3-hydroxyacyl-CoA dehydratase 3 OS=Homo sapiens GN=PTPLAD1 PE=1 SV=2
Signal recognition particle receptor subunit beta OS=Homo sapiens GN=SRPRB PE=1 SV=3
Signal peptidase complex catalytic subunit SEC11A OS=Homo sapiens GN=SEC11A PE=1 SV=1
Stromal cell-derived factor 2-like protein 1 OS=Homo sapiens GN=SDF2L1 PE=1 SV=2
GH3 domain-containing protein OS=Homo sapiens GN=GHDC PE=1 SV=2
Syntaxin-8 OS=Homo sapiens GN=STX8 PE=1 SV=2
Dolichol-phosphate mannosyltransferase subunit 3 OS=Homo sapiens GN=DPM3 PE=1 SV=2

Species	Peptide	Lys6	Lys8	Organelle
HUMAN	43	1.5147	5.0894	ER
HUMAN	33	1.6852	11.557	ER
HUMAN	25	0.9387	15.9431	ER
HUMAN	20	0.7965	2.4845	ER
HUMAN	32	1.2142	0.8964	ER
HUMAN	19	1.0002	0.3839	ER
HUMAN	16	ND	ND	ER
HUMAN	13	1.5868	0.3839	ER
HUMAN	24	1.0717	0.3839	ER
HUMAN	17	1.2835	27.1939	ER
HUMAN	10	1.483	ND	ER
HUMAN	7	0.1393	0.3839	ER
HUMAN	9	0.9903	ND	ER
HUMAN	6	1.3674	21.4636	ER
HUMAN	13	0.8023	10.6105	ER
HUMAN	7	ND	ND	ER
HUMAN	9	1.3323	11.4973	ER
HUMAN	8	1.6828	ND	ER
HUMAN	6	1.1434	16.7416	ER
HUMAN	6	ND	ND	ER
HUMAN	6	1.0487	10.9698	ER
HUMAN	6	ND	ND	ER
HUMAN	5	ND	ND	ER
HUMAN	5	ND	ND	ER
HUMAN	5	ND	ND	ER
HUMAN	7	1.329	6.9083	ER
HUMAN	6	0.0121	0.3839	ER
HUMAN	4	1.302	7.9807	ER
HUMAN	4	ND	ND	ER
HUMAN	2	ND	ND	ER
HUMAN	3	ND	ND	ER
HUMAN	3	ND	ND	ER
HUMAN	2	ND	ND	ER
HUMAN	5	ND	ND	ER
HUMAN	3	ND	ND	ER
HUMAN	2	0.0121	0.3839	ER
HUMAN	2	1.3415	0.3839	ER
HUMAN	4	ND	ND	ER
HUMAN	4	ND	ND	ER
HUMAN	2	ND	ND	ER
HUMAN	2	ND	ND	ER
HUMAN	2	0.7796	0.3839	ER
HUMAN	3	ND	ND	ER
HUMAN	2	2.0374	ND	ER
HUMAN	2	ND	ND	ER
HUMAN	2	ND	ND	ER
HUMAN	3	ND	ND	ER
HUMAN	3	ND	ND	ER
HUMAN	2	ND	ND	ER

HUMAN	2	ND	ND	ER
HUMAN	3	ND	ND	ER
HUMAN	2	1.9573	0.3839	ER
HUMAN	2	ND	ND	ER
HUMAN	3	ND	ND	ER
HUMAN	2	ND	ND	ER
HUMAN	2	ND	ND	ER
HUMAN	2	0.0121	0.3839	ER
HUMAN	2	ND	ND	ER
HUMAN	2	0.0121	0.3839	ER
HUMAN	2	ND	ND	ER
HUMAN	3	ND	ND	ER
HUMAN	2	ND	ND	ER
HUMAN	2	1.2062	9.0175	ER
HUMAN	2	ND	ND	ER
HUMAN	2	ND	ND	ER
HUMAN	1	1.2078	21.7272	ER
HUMAN	1	ND	ND	ER
HUMAN	1	ND	100	ER
HUMAN	1	ND	ND	ER
HUMAN	1	ND	100	ER
HUMAN	2	ND	ND	ER
HUMAN	3	ND	ND	ER
HUMAN	2	ND	ND	ER
HUMAN	2	ND	ND	ER
HUMAN	1	ND	ND	ER
HUMAN	1	ND	ND	ER
HUMAN	1	ND	ND	ER
HUMAN	1	100	ND	ER
HUMAN	1	ND	ND	ER
HUMAN	1	ND	ND	ER
HUMAN	2	ND	ND	ER
HUMAN	1	0.0114	0.1042	ER
HUMAN	1	1.212	ND	ER
HUMAN	1	ND	100	ER
HUMAN	1	ND	ND	ER
HUMAN	1	ND	ND	ER
HUMAN	1	ND	ND	ER
HUMAN	1	ND	ND	ER
HUMAN	1	ND	ND	ER
HUMAN	1	ND	ND	ER
HUMAN	1	ND	ND	ER
HUMAN	1	ND	ND	ER
HUMAN	1	ND	ND	ER
HUMAN	1	ND	ND	ER
HUMAN	1	ND	ND	ER
HUMAN	1	ND	ND	ER
HUMAN	1	ND	ND	ER
HUMAN	1	0.0121	0.3839	ER
HUMAN	1	ND	ND	ER

HUMAN	1	ND	ND	ER
HUMAN	1	ND	ND	ER
HUMAN	1	ND	ND	ER
HUMAN	1	ND	ND	ER
HUMAN	1	ND	ND	ER
HUMAN	0	ND	ND	ER
HUMAN	1	ND	ND	ER
HUMAN	1	ND	ND	ER
HUMAN	1	ND	ND	ER
HUMAN	1	ND	ND	ER
HUMAN	1	ND	ND	ER
HUMAN	1	1.1361	3.0501	ER
HUMAN	1	ND	ND	ER
HUMAN	1	ND	ND	ER
HUMAN	0	1.1991	37.9053	ER
HUMAN	1	ND	ND	ER
HUMAN	1	ND	ND	ER
HUMAN	1	ND	ND	ER
HUMAN	1	ND	ND	ER
HUMAN	0	ND	ND	ER
HUMAN	1	ND	ND	ER
HUMAN	1	ND	ND	ER
HUMAN	0	0.0121	0.3839	ER
HUMAN	0	ND	ND	ER
HUMAN	0	ND	ND	ER
HUMAN	0	0.0814	0.3839	ER
HUMAN	0	ND	ND	ER
HUMAN	0	ND	ND	ER
HUMAN	0	ND	ND	ER
HUMAN	0	ND	ND	ER
HUMAN	0	ND	ND	ER
HUMAN	0	ND	ND	ER
HUMAN	0	ND	ND	ER
HUMAN	0	ND	ND	ER

LIPID CLASS	PRECURSOR ION	MS/MS MODE & CONDITIONS (CE)	FRAGMENT
PG, PA	$[M-H]^-$	PI, m/z 153, -65 V	Glycerol phosphate derivative
PI	$[M-H]^-$	PI, m/z 241.1, -60 V	Cyclic inositol phosphate
PIP	$[M-H]^-$	PI, m/z 321.1, -60V	Phosphoinositol phosphate
PIP_2	$[M-H]^-$	PI, m/z 401.1, -62 V	Diphosphoinositol phosphate
PS	$[M-H]^-$	NL, m/z 185, 30 V	Serine
PE	$[M-H]^-$	PI, m/z 141, 25 V	Glycerol phosphoethanolamine derivative
PC, SM	$[M-H]^+$	PI, m/z 184.1, 60 V	Phosphocholine

SUPPLEMENTARY TABLE 2

Table-S3 Peak area and conversion into concentration of ER phospholipids infected either with H37Ra or H37Rv by shotgun lipidomics method

Peak Area				Peak Area				Peak Area			
	H37Ra				H37Rv				Uninfected (THP-1)		
**Name of Phospholipids**	**I**	**II**	**III**	**Name of Phospholipids**	**I**	**II**	**III**	**Name of Phospholipids**	**I**	**II**	**III**
PG	4301842	3453380	2391753.5	PG	4207123	5359718.9	6051295.4	PG	8464106.2	7982341	8267342
PA	2042881	1914932	1279806	PA	3383390	3833410.3	6652224.6	PA	5841275.6	6023521	5784516
PI	3624869	4280439	847382.4	PI	2825902	6779216.8	4475488.8	PI	26479545	23875934	25897616
PIP2	890.3	901.2	910.2	PIP2	5738.8	5698.2	5703.2	PIP2	1021.1	998.3	1002.4
PE	770987.5	769989.3	759899.1	PE	4676556	1631887.9	242479.9	PE	2729520.2	2698652	2703256
PS	643367.9	452969.7	427124.9	PS	590171.9	700834.7	125500	PS	688007.9	679814.3	680414.2
PC	72609876	72662858	72643589	PC	64479785	99606619	88007999	PC	37793662	37906432	37603248
SM	26959977	7170621	5172672.1	SM	5532337	9669694.5	1001176.8	SM	2532129.1	2505341	2499677

Concentration					Concentration					Concentration				
	H37Ra					H37Rv					UI			
	nmoles					nmoles					nmoles			
**Name of Phospholipids**	**I**	**II**	**III**	Average	**Name of Phospholipids**	**I**	**II**	**III**	Average	**Name of Phospholipids**	**I**	**II**	**III**	Average
PG	0.05	0.04	0.03	0.04	PG	0.05	0.06	0.07	0.06	PG	0.10	0.09	0.10	0.10
PA	3.05	2.85	1.91	2.60	PA	5.04	5.72	9.92	6.89	PA	8.71	8.98	8.63	8.77
PI	0.02	0.02	0.00	0.01	PI	0.01	0.03	0.02	0.02	PI	0.12	0.11	0.12	0.11
PIP2	0.00	0.00	0.00	0.00	PIP2	0.00	0.00	0.00	0.00	PIP2	0.00	0.00	0.00	0.00
PE	0.00	0.00	0.00	0.00	PE	0.01	0.00	0.00	0.01	PE	0.01	0.01	0.01	0.01
PS	0.04	0.03	0.03	0.03	PS	0.04	0.05	0.01	0.03	PS	0.04	0.04	0.04	0.04
PC	0.05	0.05	0.05	0.05	PC	0.04	0.07	0.06	0.06	PC	0.03	0.03	0.03	0.03
SM	4.69	1.24	0.90	2.28	SM	0.96	1.68	0.17	0.94	SM	0.44	0.44	0.43	0.44

**Name of Standard phospholipids**	**Run at Concentration**	**Peak Area**
PG	1uM	3600000
PA	1uM	28000
PI	1uM	9300000
PIP2	20uM	30000000
PE	1uM	16000000
PS	1uM	640000
PC	1uM	61000000
SM	0.5uM	120000

Supplementary Table-3

Supplementary Table-4 : PCR primers used for mRNA expression analysis

Primers	Sequence	Product size
CANX	F- ATGGATGGGGCCTGAAGAAA	225
	R- CCTCTTCCTTCACATCCGGT	
PDIA4	F- CGCGAGTTTGTCACTGCTTTC	141
	R- CGTCCTTCTTGGGGTCCATC	
ERLN2	F- CTTGTGGGACTAGTGGTGGT	154
	R-TGGTTCAGTTCGTGGTGGAT	
